# Effect of tolvaptan on renal water and sodium excretion and blood pressure during nitric oxide inhibition: a dose-response study in healthy subjects

**DOI:** 10.1186/s12882-017-0501-1

**Published:** 2017-03-13

**Authors:** Safa Al Therwani, Jeppe Bakkestrøm Rosenbæk, Frank Holden Mose, Jesper Nørgaard Bech, Erling Bjerregaard Pedersen

**Affiliations:** University Clinic in Nephrology and Hypertension, Department of Medical Research, Holstebro Hospital, Aarhus University, Hospital Unit Jutland West, Laegaardvej 12, 7500 Holstebro, Denmark

**Keywords:** Tolvaptan, Nitric oxide, ENaC, AQP2, Blood pressure, Vasoactive hormones

## Abstract

**Background:**

Tolvaptan is a selective vasopressin receptor antagonist. Nitric Oxide (NO) promotes renal water and sodium excretion, but the effect is unknown in the nephron’s principal cells. In a dose-response study, we measured the effect of tolvaptan on renal handling of water and sodium and systemic hemodynamics, during baseline and NO-inhibition with L-NMMA (L-NG-monomethyl-arginine).

**Methods:**

In a randomized, placebo-controlled, double blind, cross over study, 15 healthy subjects received tolvaptan 15, 30 and 45 mg or placebo. L-NMMA was given as a bolus followed by continuous infusion during 60 min. We measured urine output (UO), free water clearance (C_H2O_), fractional excretion of sodium (FE_Na_), urinary aquaporin-2 channels (u-AQP2) and epithelial sodium channels (u-ENaCγ), plasma vasopressin (p-AVP) and central blood pressure (cBP).

**Results:**

During baseline, FE_Na_ was unchanged. Tolvaptan decreased u-ENaC_γ_ dose-dependently and increased p-AVP threefold, whereas u-AQP2 was unchanged. During tolvaptan with NO-inhibition, UO and C_H2O_ decreased dose-dependently. FE_Na_ decreased dose-independently and u-ENaC_γ_ remained unchanged. Central BP increased equally after all treatments.

**Conclusions:**

During baseline, fractional excretion of sodium was unchanged. During tolvaptan with NO-inhibition, renal water excretion was reduced dose dependently, and renal sodium excretion was reduced unrelated to the dose, partly via an AVP dependent mechanism. Thus, tolvaptan antagonized the reduction in renal water and sodium excretion during NO-inhibition. Most likely, the lack of decrease in AQP2 excretion by tolvaptan could be attributed to a counteracting effect of the high level of p-AVP.

**Trial registration:**

Clinical Trial no: NCT02078973. Registered 1 March 2014.

**Electronic supplementary material:**

The online version of this article (doi:10.1186/s12882-017-0501-1) contains supplementary material, which is available to authorized users.

## Background

Water and salt balance in the body is a result of complex interactions of hormones, especially vasopressin and the components in the renin-angiotensin-aldosterone system. Vasopressin sensitive aquaporin2 water channels and epithelial sodium channels are expressed in the principal cells in the collecting ducts [1–3]. Stimulation of vasopressin receptors (V2) facilitates renal absorption of water and sodium [4–7]. This mechanism is antagonized by tolvaptan, a selective V2 receptor antagonist. NO may affect the renal urine concentration mechanism by membrane insertion of AQP2 in the collecting ducts principal cells [8–11]. However, it is debated whether NO inhibits or stimulates AQP-mediated water transport. In a previous study, we showed that NO promotes water excretion by a partly AVP dependent mechanism [12]. NO is synthesized from L-arginine by NO-synthase, an enzyme that is competitively inhibited by L-NMMA [13]. Systemic NO inhibition causes reduction in diuresis, natriuresis, and increases in blood pressure [14–16]. Several studies have documented the effect of selective V2 receptor antagonism on renal sodium and water excretion, but whether NO is involved in the response of the V2 receptors in the renal tubular and vascular function is not clarified. Recently, tolvaptan has been approved by the EMA for treatment of autosomal dominant polycystic kidney disease [17–21]. Also, tolvaptan is effective in conditions of dilutional hyponatremia, associated with congestive heart failure, cirrhosis and the syndrome of inappropriate secretion of antidiuretic hormone (SIADH) [22–26]. However, The European guideline on hyponatremia recommends against the use of tolvaptan in the clinical management of SIADH [27]. Thus, based on a most likely wide spread use of tolvaptan in the future, an in-depth knowledge is necessary regarding the effect of the tolvaptan on renal tubular function, vasoactive hormones and blood pressure. Our laboratory has previously investigated the effect of tolvaptan 15 mg at baseline and during tolvaptan with NO-inhibition in a randomized, placebo-controlled, double-blinded, crossover study [12]. We demonstrated a clear effect of tolvaptan 15 mg at baseline and during tolvaptan with NO-inhibition on renal water excretion and the activity in ENaC, but no knowledge is available about the effect of different doses regarding interaction between tolvaptan and NO-inhibition.

In the present study, we hypothesized 1. that tolvaptan increases water and sodium excretion in a dose-dependent manner, 2. that systemic NO-inhibition reduces this response dose-dependently, and 3. An increase in blood pressure and vasoactive hormones at baseline and during NO-inhibition counteracts tolvaptan’s effect on the tubular sodium and water transport at least partially.

The purpose was to measure the effect tolvaptan on 1. glomerular filtration rate and renal absorption of water and sodium (GFR (^51^Cr-EDTA-clearance), urinary output (UO), free water clearance (C_H2O_), fractional excretion of sodium (FE_Na_), urinary excretion of aquaporin 2 (u-AQP2), and urinary excretion of a protein fragment of the epithelial sodium channel (u-ENaC_γ_)_,_ 2.blood pressure (brachial blood pressure (Bbp), central BP (cBP), pulse wave velocity (PW), augmentation index (AI)), and 3. vasoactive hormones in plasma(vasopressin (p-AVP), angiotensinII (p-AngII), renin (PRC), and aldosterone (p-Aldo)), both at baseline and during tolvaptan with NO-inhibition in a randomized, placebo-controlled, double-blinded, crossover dose-response study of healthy volunteers.

## Methods

### Subjects

#### Inclusion criteria

Healthy non-smoking men and women, age between 18 and 40 year. and with BMI between 18.5 and 30 kg/m^2^ were enrolled.

#### Exclusion criteria

Exclusion criteria were; arterial hypertension (bBP > 140 mmHg systolic and/or >90 mmHg diastolic), history or clinical signs of neoplastic disease or disease of the heart, lungs, kidneys or endocrine organs, drug or alcohol abuse (I.e. > 14 units a week for women and 21 for men), medical treatment except oral contraceptives, pregnancy or breast-feeding, significant laboratory abnormalities in the screening test of blood samples (I.e. abnormal hemoglobin, white cell count, plasma sodium, plasma potassium, plasma creatinine, plasma alanine aminotransferase or serum cholesterol, plasma bilirubin and plasma albumin, and urine samples (I.e. albuminuria or glucosuria), abnormal electrocardiogram, blood donation less than 1 month prior to the study.

In fertile women contraceptive treatment must be used during and after the study period (I.e. oral contraceptives, spiral, depot injection of gestagen, sub dermal implantation, transdermal contraceptive patch and hormonal contraceptive vaginal ring).

Withdrawal criteria were development of one or more of the exclusion criteria, brachial blood pressure increase above 180/105 mmHg during infusion of L-NMMA, withdrawal of consent or lack of compliance.

### Study design

The trial was performed as a randomized, double-blinded, placebo- controlled, crossover, dose-response study in healthy subjects. The study consisted of four treatment periods, placebo or tolvaptan 15, 30 and 45 mg, with an intermediate wash- out period of at least 3 weeks to eliminate any carryover effects.

### Medications

Tolvaptan (SAMSCA®, Otsuka, Tokyo, Japan) 15, 30 and 45 mg or placebo were coated in identical gelatine capsules and were orally administered at 8:00 AM.

L-NMMA (Bachem, Weil am Rhein, Germany) was dissolved in isotonic saline solution and given intravenously at 11:00 AM.

### Number of subjects

Free water clearance (C_H2O_) was used as the main effect variable. With a minimal relevant difference of 6 ml/min with an estimated standard deviation (SD) of 4 ml/min, 12 subjects were needed using a level of significance of 5% and a statistical power of 90%. Due to possible drop outs 15 subjects were included.

### Recruitment

Healthy volunteers were recruited by advertising in public institutions and private companies.

### Effect variables

C_H2O_ was the primary effect variable_._ The secondary effect variables were 1) renal function (^51^Cr-EDTA-clearance, UO, u-AQP2, u-ENaC_γ_, FE_Na_), 2) hemodynamics (bBP, cBP, PWV, AI and 3) vasoactive hormones (PRC, p-ANG II, p-Aldo, p- AVP).

### Diet

Four days prior to each treatment period subjects consumed a standardized diet of 11.000 KJ day^−1^. The diet was delivered from our facilities and was composed of 15% proteins, 55% carbohydrates and 30% fat following general dietary guidelines. The sodium content was 150 mmol day^−1^. No additional sodium or other spices was allowed. The daily fluid intake was 2.5 L including a maximum of two cups of coffee or tea. No alcohol or soft drink was allowed during the 4-day diet.

### Experimental procedure

A 24-h urine was collected and a fasting period of 8 h was performed prior to each examination day. The four examinations were conducted at our facility from 7:45 AM to 1:00 PM. The procedures were identical on all examination days.

At 8:00 AM subjects were given placebo or tolvaptan 15, 30 and 45 mg. An intravenous catheter was placed in each arm to collect blood samples and infuse ^51^Cr-EDTA.

At 8:00 AM and every 30 min, an oral water load of 175 ml was given.

BP was measured every 30 min from 8:30 AM to 1:00 PM. Based on results from a dose-finding study made by our laboratory in healthy subjects [15], a bolus of L-NMMA 4.5 mg/kg was given at 11:00 AM, followed by continuous infusion (3 mg/kg/h) during 60 min. During infusion of L-NMMA BP was measured every 5 min, and every 15 min after infusion of L-NMMA.

Blood samples were drawn every 30 min from 8:30 to 1:00 PM and were analyzed for p-Na, p-osm, p-creatinine, p-albumin and p-^51^Cr-EDTA. Every 60 min; at 11:00 AM (baseline), at 12:00 AM (after end of L-NMMA infusion), and at 1:00 PM (60 min after end of L-NMMA infusion), blood samples were drawn to measure PRC, p-Aldo, p-Ang II and p-AVP.

Every 30 min from 9:30 AM to 1:00 PM, urine samples were collected by voiding in standing or sitting position after collecting BP measurements and blood samples. Otherwise subjects were kept in a supine position in a temperature-controlled (22–25 °C) and quiet room. Baseline period was mean of the first three clearance periods. The urine samples were analyzed for osmolality, creatinine, sodium, AQP2, ENaC_γ_ and ^51^Cr-EDTA.

Applanation tonometry with SpygmoCor was performed at 10:10 AM and at11: 40 AM to measure cBP, PVW and AI.

### Measurements

#### Renal function

^51^Cr-EDTA-clearance was measured using the constant infusion clearance technique with 51Cr-EDTA as reference substance.

C_H2O_ was determined using the formula C_H2O_ = UO- C_osm_, where C_osm_ is the osmolar clearance.

Clearance (C) of substance X was calculated as C_X_ = U_X_/(P_X_ x UO), where U_X_ denotes concentration of x in urine, P_X_ denotes concentration of x in plasma, and UO is urine excretion rate.

Fractional excretion of sodium was determined according to the following formula FE_Na_ = C_Na_/^51^Cr-EDTA-clearance x100%, where C_Na_ is sodium clearance.

#### Urinary excretion of AQP2 and ENaC_γ_

Urine samples were kept frozen at −20 °C until assayed. U-AQP2 and u-ENaC_γ_ were measured by radioimmunoassay as previously described [28–30]. Antibodies were raised in rabbits to synthetic peptide as previously described [12]. U-ENaC_γ_ was measured by radioimmunoassay as previously described [29]. Antibodies were raised against the synthetic ENaC_γ_ peptide in rabbits and affinity purified as described previously [12, 31]. The anti-AQP2 antibody was a gift from Soerens Nielsen, The Water and Salt Research Center, Institute of Anatomy, Aarhus University, Denmark.

#### Vasoactive hormones in plasma

Blood samples collected for measurements of vasoactive hormones were centrifuged and plasma was separated, and kept frozen until assayed as previously described [12]. PRC was determined by immunoradiometric assay as previously described [12]. Aldo was determined by RIA as previously described [12]. Ang II and AVP were extracted from plasma and then determined by radioimmunoassay [12, 32, 33].

#### Brachial and central blood pressure

Brachial BP was measured using an oscillometer (Omron 705IT) and recorded as previously described [12]. Central BP, PWA and carotid-femoral PWV were measured using applanation tonometry (SphygmoCor® CPV system®, AtCor Medical, Sydney, Australia) as previously described [12].

#### Routine analyses

Sodium, glucose, albumin, and hemoglobin were measured by routine methods in Department of Clinical Biochemistry, Holstebro Hospital.

#### Statistics

We performed statistical analyses by using IBM SPSS statistics version 20.0.0 (SPSS Inc., Chicago, IL, USA). General Linear Model Repeated Measures was used for comparison between and within subjects to test differences between placebo and tolvaptan treatment at baseline, during and after infusion of L-NMMA. One-way ANOVA was performed for comparison between treatment groups.

To compare differences between treatment groups at baseline, paired sample *t*-test was performed during and after infusion of L-NMMA. Statistical significance was at <0.05 in all analyses. Data with normal distribution are reported as means ± SD or SEM. Non-parametric test was performed for data with non-normal distribution, and are reported as medians with 25th and 75th percentiles.

## Results

### Demographics

Twenty-seven healthy subjects were allocated to the trial. Twelve of the participants were excluded; three due to difficulties to gain intravenous access, two due to arterial hypertension and seven due to withdrawal of consent. Fifteen participants completed the study, three males and 12 females. Median age was 24 ± 2 years, weight 71.8 ± 13.1 kg, BMI 24 ± 4 kgm^2^. P-sodium 141 ± 1.7 mmol/L, p-potassium 3.7 ± 0.2 mmol/L, p-creatinine 68.7 ± 9.8 μmol/L. Systolic brachial blood pressure (SBP) 119 ± 10 mmHg, diastolic brachial blood pressure (DBP) 72 ± 8 mmHg.

### Urine collection before the examination day

Twenty-four hours urine samples were collected prior to every examination day. Values are shown in Additional file 1: Table S1. No differences were measured in UO, C_H2O_, u-AQP2, u-Na and u-K between the treatment periods.

### ^51^Cr-EDTA-clearance

During tolvaptan with NO inhibition, ^51^Cr-EDTA-clearance did not change significantly. The relative changes in ^51^Cr-EDTA-clearance were non-significant in the four treatment arms (Table 1).

**Table 1:** Effect of tolvaptan 15, 30 and 45 mg at baseline, during and after NO-inhibition on the relative changes in GFR (∆^51^ CrEDTA-clearance), urinary output (∆ UO), free water clearance (Δ C_H2O_), urinary aquaporin 2 excretion rate (∆ AQP2), fractional excretion of sodium (∆ FE_Na_) and urinary ENaCγ excretion rate (Δ ENaCγ) in a randomized, placebo-controlled, double-blind, crossover, dose–response study of 15 healthy subjects

Periods	Baseline	L-NMMA		Post infusion		p (GLM-within)
	0–90 min	90–120 min	120–150 min	150–180 min	180–210 min	
Δ ^51^Cr-EDTA-clearance (%)						
Placebo	-	−5 ± 9	−1 ± 2	−5 ± 3	−3 ± 8	0.746
Tolvaptan 15 mg	-	−1 ± 7	2 ± 4	−2 ± 5	1 ± 5	
Tolvaptan 30 mg	-	−5 ± 7	−3 ± 7	−3 ± 6	−4 ± 7	
Tolvaptan 45 mg	-	1 ± 6	1 ± 7	−0.1 ± 7	−2 ± 7	
p (GLM between)			0.447			
Δ UO (%)						
Placebo	-	−49 (−59;−13)	−41 (−49;−5)	−26 (−42;5)	−6 (−27;30)	<0.0001
Tolvaptan 15 mg	-	−46 (−57;−34)	−48 (−51;−38)	−45 (−49;−39)	−40 (−47;−26)	
Tolvaptan 30 mg	-	−40 (−54;−32)	−42 (−55;−34)	−47 (−55;−28)	−39 (−44;−25)	
Tolvaptan 45 mg	-	−31 (−38;−27)	−42 (−43;−31)	−32 (−41;−26)	−31 (−36;−24)	
p (GLM between)			0.002			
p (ANOVA)	-	0.217	0.078	0.001	<0.0001	
Δ C_H2O_ (%)						
Placebo	-	−57 (−78;−25)	−47 (−63;0)	−31 (−50;14)	−0.5 (−31;37)	<0.0001
Tolvaptan 15 mg	-	−53 (−66;−41)	−56 (−60;−39)	−54 (−66;−47)	−51 (−59;−46)	
Tolvaptan 30 mg	-	−38 (−65;−33)	−46 (−66;−38)	−51 (−63;−35	−42 (−61;−31)	
Tolvaptan 45 mg	-	−35 (−49;−30)	−47 (−57;−36)	−33 (−54;−30)	−31 (−46;−26)	
p (GLM between)			0.004			
p (ANOVA)	-	0.156	0.130	0.011	0.002	
Δ AQP2 (%)						
Placebo	-	−19 (−29;−2)	−21 (−29;−18)	−21 (−28;−12)	−13 (−23;−3)	0.233
Tolvaptan 15 mg	-	−18 (−26;−7)	−13 (−18;−7)	−9 (−18;−2)	−4 (−18;11)	
Tolvaptan 30 mg	-	−14 (−26;−5)	−19 (−28;−11)	−12 (−24;−2)	−4 (−13;6)	
Tolvaptan 45 mg	-	−9 (−22;3)	−13 (−22;−2)	−13–23;−4) (	−2 (−11;6)	
p (GLM between)			0.094			
ΔFE_Na_ (%)						
Placebo	-	−25 (−33;−9)	−33 (−40;−28)	−12 (−30;1)	4 (−11;25)	0.433
Tolvaptan 15 mg	-	−14 (−28;−3)	−17 (−23;8)	17 (−5;34)	46 (0;55)	
Tolvaptan 30 mg	-	−15 (−18;9)	−10 (−18;9)	13 (0;19)	44 (19;61)	
Tolvaptan 45 mg	-	−12 (−28;4)	−9 (−26;4)	3 (11;64)	45 (19;91)	
p (GLM between)			0.014			
p (ANOVA)	-	0.207	0.009	0.014	0.021	
Δ ENaC_γ_ (%)						
Placebo	-	−11 (−16;−7)	−8 (−20;−3)	−11 (−23;−3)	2 (−13;18)	
Tolvaptan 15 mg	-	−9 (−21;−2)	−7 (−15;22)	5 (−8;13)	6 (−7;−21)	
Tolvaptan 30 mg	-	−3 (−13;3)	−5 (−10;5)	3 (−1; 13)	8 (−8;19)	
Tolvaptan 45 mg	-	−13 (−21;3)	−5 (−10;6)	−2 (−8;10)	13 (−7;18)	
p (Friedman)	-	0.564	0.392	0.008	0.031	

Data are presented as mean ± SD or medians with 25 ^th^ and 75 ^th^ percentiles in parentheses. General linear model (GLM) with repeated measures was performed for comparison within and between groups or Friedman test for comparison between groups. One-way ANOVA was used to test differences between tolvaptan 15, 30 and 45 mg vs placebo. Paired *t*-test was used for comparison between the three tolvaptan doses at baseline period (0–90 min), L-NMMA period (90–150 min) and post infusion period (150–210 min); the significance levels are listed under the result section

### Urinary output and free water clearance

**Fig. 1:**
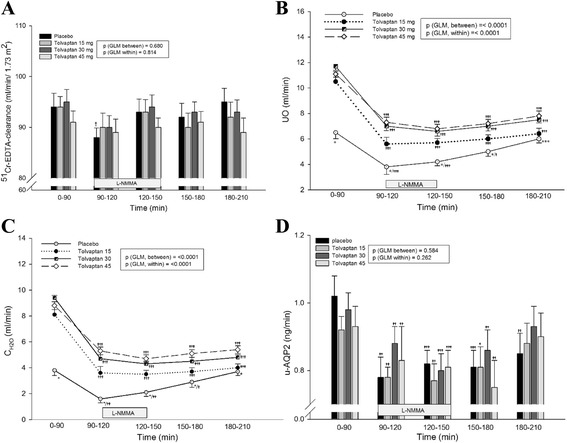
Effect of tolvaptan 15, 30 and 45 mg at baseline, during and after NO-inhibition on GFR (^51^ Cr-EDTA-clearance) (**a**), UO (**b**), C_H2O_ (**c**) and u-AQP2 (**d**). Data are presented as mean ± SEM. General linear model (GLM) with repeated measures was performed for comparison within and between groups. One-way ANOVA (*) was used to test differences between tolvaptan 15, 30 and 45 mg vs placebo. Paired *t*-test (α/β/γ) was used for comparison of infusion period (90–150 min) vs baseline period (0–90 min) and post infusion period (150–210 min) vs baseline period.**†** *p*<; 0.05; **††** *p* < 0.001; */**†††** *p* < 0.0001. Paired *t*-test was used for comparison between the three tolvaptan doses at baseline period (0–90 min), L-NMMA infusion period (90–150 min) and post infusion period (150–210 min); the significance levels are listed under the result section

Absolute and relative values of UO and C_H2O_ are shown in Fig. 1b-c and Table 1.

During L-NMMA infusion 90–120 min, UO decreased significantly during all treatments (placebo: 3.0 ml/min (49%); tolvaptan 15 mg: 4.8 ml/min (46%); tolvaptan 30 mg: 4.6 ml/min (40%); tolvaptan 45 mg: 3.6 ml/min (31%)). As during baseline, the absolute level of UO remained lower during placebo than tolvaptan, and no significant differences were measured between the different doses of tolvaptan. The relative changes from baseline to L-NMMA infusion were the same during both placebo and tolvaptan treatment. However, a tendency to a dose response effect was seen both in the absolute and relative measurements. During the post infusion period 180–210 min, UO increased during placebo to the same level as during baseline, but remained reduced to the same levels during the three doses of tolvaptan (placebo: 0.7 ml/min (6%); tolvaptan 15 mg: 4.1 ml/min (40%); tolvaptan 30 mg: 4.6 ml/min (39%); tolvaptan 45 mg: 3.1 ml/min (31%)).

During L-NMMA infusion 90–120 min, C_H2O_ decreased during all treatments (placebo: 2.1 ml/min (57%); tolvaptan 15 mg: 4.3 ml/min (53%); tolvaptan 30 mg: 4.2 ml/min (38%); tolvaptan 45 mg: 3.3 ml/min (35%)). The absolute level of C_H2O_ remained lower during placebo than tolvaptan treatment, and C_H2O_ decreased significantly more after tolvaptan 15 mg compared to 30 and 45 mg (*p* = 0.034 and 0.004 respectively). The relative changes from baseline to L-NMMA infusion did not deviate significantly between the four treatments, but a tendency to a dose response effect was measured with the lowest decrease in the highest tolvaptan dosis. During the post infusion period 180–210 min, C_H2O_ increased during placebo to the same level as during baseline, but remained reduced during tolvaptan treatment (placebo: 0.5 ml/min (0.5%); tolvaptan 15 mg: 4.2 ml/min (51%)); tolvaptan 30 mg: 4.2 ml/min (42%); tolvaptan 45 mg: 3.2 ml/min (31%)). The difference in C_H2O_ between the three tolvaptan doses was non-significant but a tendency to a dose response effect was seen.

### Fractional excretion of sodium

**Fig. 2:**
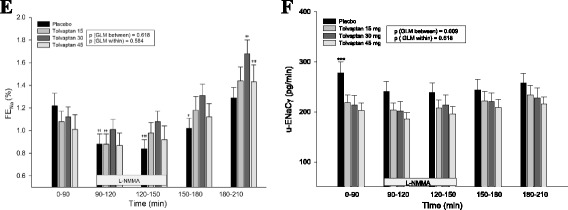
Effect of tolvaptan 15, 30 and 45 mg at baseline, during and after NO-inhibition on FE_Na_ (**e**) and u-ENaCγ (**f**). Data are presented as mean ± SEM. General linear model (GLM) with repeated measures was performed for comparison within and between groups. One-way ANOVA (*) was used to test differences between tolvaptan 15, 30 and 45 mg vs placebo. Paired *t*-test (α/β/γ) was used for comparison of infusion period (90–150 min) vs baseline period (0–90 min) and post infusion period (150–210 min) vs baseline period. **†** *p*<; 0.05; **††** *p* < 0.001; */**†††** *p* < 0.0001. Paired *t*-test was used for comparison between the three tolvaptan doses at baseline period (0–90 min), L-NMMA infusion period (90–150 min) and post infusion period (150–210 min); the significance levels are listed under the result section

Absolute and relative values of FE_Na_ are shown in Fig. 2e and Table 1.

During L-NMMA infusion 90–120 min, FE_Na_ decreased significantly after placebo and tolvaptan 15 mg (placebo: 0.37% (the relative decrease in percent: 25%); tolvaptan 15 mg: 0.20% (14%); tolvaptan 30 mg: 0.12% (15%); tolvaptan 45 mg: 0.14% (12%)). However, FE_Na_ decreased to the same level as after tolvaptan 30 and 45 mg during the first 30 min of L-NMMA infusion period 90–120 min. However, during the last LNMMA infusion period 120–150 min, FE_Na_ decreased to a significantly lesser extent after all tolvaptan doses compared with placebo (Table 1). During the post infusion period 180–210 min, FE_Na_ increased after all treatments, but to a much higher level after tolvaptan (placebo: 0.04% (the increase in percent; 4%); tolvaptan 15 mg: 0.36% (46%); tolvaptan 30 mg:0.52% (44%); tolvaptan 45 mg: 0.42% (45%)). No dose response pattern was seen either in the absolute or relative measurements of FE_Na_.

### U-AQP2 and U-ENaC_γ_

During tolvaptan with NO-inhibition 90–120 min, u-AQP2 decreased to the same level within all treatment arms. The same response was observed in the relative changes. During the post infusion period 180–210 min, u-AQP2 remained reduced during placebo treatment, but had increased to baseline level during all tolvaptan doses. No dose response pattern was seen, in either the absolute or relative measurements of u-AQP2 (Fig. 1d and Table 1).

During tolvaptan with NO-inhibition 90–120 min, u-ENaC_γ_ decreased significantly after placebo, but during tolvaptan treatment no significant differences existed between the placebo group and the three tolvaptan doses either in absolute or relative measurements (Fig. 2f and Table 1). During the post infusion period 180–210 min, u-ENaC_γ_ was unchanged compared to baseline measurement both during placebo and tolvaptan treatment. A significant difference was measured in the relative values with a dose response pattern and the highest increase during the highest tolvaptan dosis (Table 1).

### Plasma sodium and plasma osmolarity

At baseline, p-Na and p-osm were significantly higher and similar after tolvaptan 15, 30 and 45 mg compared to placebo and this response was sustained during tolvaptan with NO-inhibition (Additional file 2: Table S2).

### Vasoactive hormones

During tolvaptan with NO-inhibition, none of the vasoactive hormones were affected a part from the sustained 3- fold increase in p-AVP after all three tolvaptan doses (Additional file 3: Table S3).

### Brachial blood pressure

During baseline conditions, SBP, DBP and pulse rate were the same within all treatments (Additional file 4: Table S4).

In response to tolvaptan with NO-inhibition, DBP increased after placebo, but remained constant after tolvaptan 15, 30 and 45 mg. Otherwise no differences were measured in SBP and pulse rate.

### Central blood pressure

At baseline, PWV, AI, CSBP and CDBP were identical after tolvaptan 15, 30 and 45 mg versus placebo. During tolvaptan with NO-inhibition, CSBP and CDBP increased equally after all treatments (Table 2). PWV was not affected by L-NMMA infusion after placebo whereas a similar increase after tolvaptan 15, 30 and 45 mg was measured, but only to the same level observed after placebo. Tolvaptan 15 and 30 mg did not change AI. The value of AI was higher after tolvaptan 45 mg. However, no differences were measured between treatment groups.

**Table 2:** Effect of tolvaptan 15, 30 and 45 mg at baseline, during and after NO-inhibition on pulse wave velocity (PWV), augmentation index (AI), central diastolic and systolic blood pressure (CBDP and CSBP) in a randomized, placebo-controlled, double-blind, crossover, dose-response study of 15 healthy subjects

	Prior to L-NMMA infusion (at 70 min)	During L-NMMA infusion (at 130 min)
PWV(m/s)		
Placebo	5.2 ± 0.5	5.4 ± 0.5
Tolvaptan 15 mg	5.0 ± 0.5	5.4 ± 0.6^**^
Tolvaptan 30 mg	5.2 ± 0.6	5.5 ± 0.6 ^**^
Tolvaptan 45 mg	5.2 ± 0.3	5.5 ± 0.4^**^
p (ANOVA)	0.518	0.851
AI		
Placebo	−0.7 ± 13.8	0.1 ± 15.1
Tolvaptan 15 mg	−1.5 ± 12.5	−1.2 ± 17.3
Tolvaptan 30 mg	−0.4 ± 11.8	2.5 ± 17.8
Tolvaptan 45 mg	−3.3 ± 15.8	1.6 ± 12.0^*^
p (ANOVA)	0.968	0.961
CSBP		
Placebo	97 ± 9	100 ± 8^**^
Tolvaptan 15 mg	97 ± 9	101 ± 10^*^
Tolvaptan 30 mg	96 ± 9	102 ± 9^**^
Tolvaptan 45 mg	96 ± 8	102 ± 6^**^
p (ANOVA)	0.989	0.952
CDBP		
Placebo	63 ± 7	68 ± 6^***^
Tolvaptan 15 mg	63 ± 7	68 ± 9^**^
Tolvaptan 30 mg	61 ± 6	68 ± 7^***^
Tolvaptan 45 mg	62 ± 5	68 ± 6^**^
p (ANOVA)	0.936	0.998

Data are presented as mean ± SD. One-way ANOVA was performed to test differences between treatments groups. Paired *t*-test (*) was used for comparison of data during L-NMMA infusion vs prior to L-NMMA infusion

*p < 0.05; **p < 0.001; ***p < 0.0001

### Water and sodium excretion, BP and vasoactive hormones during baseline

^51^Cr-EDTA-clearance was similar after placebo and tolvaptan (Fig. 1a).

UO was significantly lower during placebo than tolvaptan treatment (Fig. 1b). UO was lower after tolvaptan 15 mg compared with tolvaptan 30 mg (*p* = 0.005), while no difference was measured between tolvaptan 30 and 45 mg. C_H2O_ was significantly lower during placebo than tolvaptan treatment. C_H2O_ increased significantly more after tolvaptan 30 mg compared with 15 mg (*p* = 0.004), but no further increase was measured after tolvaptan 45 mg (Fig. 1c).

FE_Na_ was the same after all treatments. No significant difference was measured between the three tolvaptan doses (Fig. 2e).

U-AQP2 was similar after tolvaptan 15, 30 and 45 mg or placebo (Fig. 1d). U-ENaC_γ_ was significantly higher after placebo than after tolvaptan treatment, and a dose-response effect was measured with the lowest level of u-ENaCγ during the highest tolvaptan dosis (Fig. 2f).

**Fig. 3:**
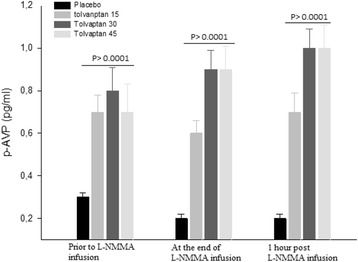
Effect of tolvaptan 15, 30 and 45 mg on p-AVP at baseline, during and after NO-inhibition. Data are depicted as mean ± SEM. Friedman test was used for comparison between treatment groups prior to L-NMMA infusion, at the end of L-NMMA infusion and 1 h after the end of L-NMMA infusion

PRC, p-ANGII and p-Aldo were the same after tolvaptan 15, 30 and 45 mg compared with placebo (Additional file 3: Table S3). A highly significant and non-dose dependent 3-fold increase in p-AVP was measured after all three doses of tolvaptan (Fig. 3). No difference was measured between the three tolvaptan doses (*p* = 0.645).

## Discussion

In the present dose-response study, we investigated short-term effects of tolvaptan on renal tubular function, vasoactive hormones and systemic hemodynamics during basal conditions and during tolvaptan with NO-inhibition in healthy subject. The present study is a continuation and expansion of a previous randomized, double-blinded, placebo-controlled crossover, single-dose study conducted by our laboratory [12].

At baseline, tolvaptan increased renal water excretion dose-dependently up to 30 mg, without affecting sodium excretion. During L-NMMA infusion, tolvaptan decreased water excretion dose-dependently, whereas sodium excretion decreased independently of the dose. In the post infusion period, water excretion remained reduced during all tolvaptan doses, but increased during placebo. Tolvaptan increased sodium excretion to a higher level.

Recent dose-response trials have shown that oral administration of tolvaptan 30 mg led to a fast increase in the effective concentration in plasma. This increase was however followed by a fast decline due to tolvaptan’s short half-life of approximately 6–8 h [34, 35]. The absolute bioavailability of tolvaptan is approximately 56% and a plasma concentration peak (300 ng/ml) is obtained 2–4 h post-dosing. Based on the pharmacological profile of tolvaptan, we think that oral administration of tolvaptan at 8:00 AM (approximately 3 h prior to L-NMMA infusion) was the optimal time. The results from our previous study of tolvaptan 15 mg confirmed a significant increase in OU and C_H2O_ that was measurable within approximately 2-3 h post-dose [12].

In the present trial, tolvaptan increased UO and C_H2O_ dose-dependently when tolvaptan dose was ascended from 15 to 30 mg during baseline, but no further increase was measured after tolvaptan 45 mg. This observation does not exclude that a much higher dose might further increase renal water excretion. In addition, a longer observation time might be needed to obtain a further increase in UO and C_H2O._ Several of the participants reported significant and frequent diuresis after they have left our facilities. In previous clinical studies, using much higher tolvaptan doses ranging from 60 to 480 mg, tolvaptan increased C_H2O_ in a dose-independent relationship and UO in a dose-dependent relationship [35, 36]. In these studies, UO and C_H2O_ were collected and measured over a longer observation period around 24-h and up to 144 h post-dose.

During L-NMMA infusion, UO and C_H2O_ declined in all treatment arms, confirming the well-known antidiuretic effect by blocking the diuretic action of NO. However, UO remained approximately 30–50% higher after tolvaptan, and p-AVP also remained at the high level and unchanged from baseline. The degree of decline in C_H2O_ tended to be dependent of the tolvaptan dose, as C_H2O_ fell less after tolvaptan 30 and 45 mg than after 15 mg. In the post infusion period, UO and C_H2O_ remained reduced to the same levels during the three tolvaptan doses. This may reflect an interaction between the V2 receptor system and the NO system, expressed as a higher level of water excretion after tolvaptan. Thus, tolvaptan partly antagonized the antidiuretic action of L-NMMA. This is in agreement with NO having a stimulating effect on renal water excretion in the renal principal cells that seemed to be partly AVP-dependent. Our results are, however, not in agreement with the findings observed in our previous clinical study of tolvaptan 15 mg, in which we showed that tolvaptan potentiated the antidiuretic action of L-NMMA [12]. A possible explanation of this discrepancy could be that tolvaptan was given 2 h earlier, at 6:00 AM versus 8:00 AM in the present study. Therefore, tolvaptan exerted its effect over a longer period before the L-NMMA intervention.

Tolvaptan prevented fusion of cytoplasmic vesicles containing AQP2 to the apical membrane as response to activation of V2 receptors by AVP [1, 2]. As a result, the activation rate of AQP2 is declined. This response is biochemically reflected as a decline in the excretion of AQP2 into the urine [28–30]. Increased u-AQP2 reflects increased water transport from the tubular lumen to the intracellular space via the aquaporin2 water channels, located in the principal cells in the distal part of the nephron [28, 30]. Surprisingly, u-AQP2 did not change after oral administration of tolvaptan, not even in response to the highest dose of tolvaptan. We measured a similar response in our previous study of tolvaptan 15 mg [12]. Most likely, this could be explained by the measured 3-fold increase in plasma AVP. The increase in AVP exerts a competitive inhibition on the V2 receptor, thereby interfering with tolvaptan’s binding to the V2 receptor. Alternatively, the baseline period could be too short to reflect the expected decline in u-AQP2 after oral administration of tolvaptan.

At baseline, FE_Na_ was identical after all treatments, whereas u-ENaC_γ_ decreased dose-independently after tolvaptan. Several experimental and clinical trials have appointed the AVP-V2R-ENaC_γ_ axis as an essential physiological mechanism in the final regulation of sodium excretion [6, 37]. Our findings are in agreement with these results, as we measured a reduced and similar baseline level of u-ENaC_γ_ after all tolvaptan doses. There was a clear tendency to a dose-response pattern with the lowest u-ENaC_γ_ at the highest dosis of tolvaptan. However, we did not measure an increase in FE_Na_. This observation could be explained by the primarily aquaretic effect of selective V2R antagonism that is shown to be approximately 4 times the natriuretic effect [6].

During tolvaptan with NO-inhibition, FE_Na_ declined less pronouncedly after tolvaptan and the degree of the decline was not dependent of tolvaptan dosis. The sodium transport via the epithelial sodium channels is supposed to be reflected by the level of urinary excretion of a protein fraction from the channels (u-ENaC_γ_) [29, 38]. U-ENaC_γ_ decreased after placebo and remained unchanged, but at a lower level after tolvaptan treatment. In the post infusion period, FE_Na_ increased more and dose-independently after tolvaptan. U-ENaC_γ_ also increased, but with a dose response relationship and the highest increase was measured during tolvaptan 45 mg. This observation was different from our previous single-dose study of tolvaptan, where u- ENaC_γ_ increased after NO-inhibition and remained higher in the post infusion period. This in turn, could also be attributed to the 2 h longer reaction time for tolvaptan in the first study [12], during which the effective concentration in plasma may have been lowered owing tolvaptan’s relatively short half-life. Therefore, a lesser effect remained when L-NMMA was administered. Thus, tolvaptan inhibited the antinatriuretic effect of systemic NO-inhibition partially via an AVP-dependent pathway, and the degree of inhibition followed a dose-independent relationship.

We measured a significant and three-fold non-dose-dependent increase in p-AVP after all three tolvaptan doses. Endogenous AVP release appeared to reach a plateau at a tolvaptan dose of 15 mg. This compensatory release of AVP due to treatment with V2 receptor antagonist is well documented [26]. However, the absence of a dose-response pattern in vasopressin has previously been demonstrated during tolvaptan treatment. Also, this response in plasma AVP was not affected by inhibition of systemic NO after all treatments. Thus, NO did not seem to play a role in the AVP releasing mechanism. Our results are in agreement with previous studies [6, 12].

Brachial and central BP remained unaffected even with an approximate 3-fold increase in p-AVP after tolvaptan 15, 30 amd 45 mg. Applanation tonometry was also performed under standardized conditions. The method is described and evaluated elsewhere [39]. Inhibition of systemic NO synthesis caused an increase in cBP, but to the same level within all treatments. In addition to its aquaretic effect, tolvaptan is believed to exert a pressor effect via the V1a receptors, localized in the vascular smooth muscle cells, as a consequence of compensatory increased endogenous AVP release. In turn, AVP may activate the V1a receptors at high plasma levels [6]. However, we demonstrated that an increase in tolvaptan dose from 15 to 45 mg had no effect on bBP and cBP. This is in good agreement with the fact that we did not measure changes in P-AngII, PRC or P-Aldo after any of the three tolvaptan doses. In other words, we could not reject the null hypothesis, saying that tolvaptan would not change blood pressure using different doses of tolvaptan.

During baseline conditions, we measured a non-dose dependent increase in p-Na and p-osm after tolvaptan. Plasma sodium and p-osm remained 141 mmol/L and 288 mosm/kg respectively, even after tolvaptan 45 mg. Most likely, the response in p-Na and p-osm must be due to increased renal water excretion and consequently decline in the extracellular volume. V2 receptor antagonism has previously been shown to increase p-Na and p-osm in a non-dose-response manner [12, 34, 36]. The abovementioned parameters were not affected by NO inhibition. The results are in agreement with a previous clinical study reported by our laboratory [12].

## Strengths and limitations

The major strengths of our study are a combination of the study design as randomized, placebo-controlled, double-blinded, crossover trial, and the standardized diet and fluid intake to avoid any confounding of the results. It might be considered as a weakness of the study that the assumption of abrogated systemic NO synthesis was exclusively based on the measurement of the response in ^51^Cr-EDTA-clearance, C_H2O_, UO, u-AQP_2_, FE_Na_ and mean arterial blood pressure. A measurement of nitrate or nitrite in plasma or urine could have documented the NO inhibition directly, but this method is difficult, and according to our experience not as reliable as the methods used in the present study.

Emerging data indicate that the regulated protein fractions of AQP2 and ENaC_γ_ are excreted as urinary exosomes. These fraction were not isolated and analyzed in the present study.

We cannot exclude possible gender differences in the parameters studied due to female predominance.

## Conclusions

During baseline conditions, tolvaptan increased renal water excretion dose-dependently, whereas tubular handling of sodium was unchanged. During tolvaptan with NO-inhibition, renal water excretion was reduced dose-dependently, and renal sodium excretion non-dose-dependently partly via an AVP-dependent mechanism. The lack of decrease in u-AQP2 by tolvaptan could be explained by a counteracting effect of elevated vasopressin in plasma.

## Additional files

Additional file 1: Table S1. Urine output, free water clearance (C_H2O_), urinary AQP2 excretion per minute (u-AQP2), urinary sodium excretion (u-Na) and urinary potassium excretion (u-K) during 24-h urine collection in a randomised, placebo-controlled, double-blind, crossover, dose-response study of 15 healthy subjects. Values are means with ± SD. One-way ANOVA was used for comparison between groups. (PDF 9 kb)
Additional file 2: Table S2. Effect of tolvaptan 15, 30 and 45 mg at baseline, during and after NO-inhibition on plasma concentration of sodium and plasma osmolality in a randomized, placebo-controlled, double-blind, crossover, dose-response study of 15 healthy subjects. Data are presented as mean ± SD. General linear model (GLM) with repeated measurements was performed for comparison within and between groups. One-way ANOVA was used to test differences between tolvaptan 15, 30 and 45 mg vs placebo. Paired *t*-test was performed for comparison of infusion period (90–150 min) vs baseline period (0–90 min), and post infusion period (150–210 min) vs baseline period. (PDF 84 kb)
Additional file 3: Table S3. Effect of tolvaptan 15, 30 and 45 mg at baseline, during and after NO-inhibition on plasma concentrations of renin (PRC), angiotensin II (P-AngII) and aldosterone (P-aldo) in a randomized, placebo-controlled, double-blind, crossover, dose-response study of 15 healthy subjects. Data are presented as mean ± SD. General linear model (GLM) with repeated measures was performed for comparison within and between groups. Paired *t*-test was used for comparison between L-NMMA infusion period (at the end of L-NMMA infusion period) vs baseline (prior to L-NMMA infusion period) and at baseline vs post infusion period (1 h after L-NMMA infusion period) vs baseline period. One-way ANOVA was performed to test differences between treatment groups. (PDF 14 kb)
Additional file 4: Table S4. Effect of tolvaptan 15, 30 and 45 mg at baseline, during and after NO-inhibition on brachial systolic blood pressure (SBP), diastolic blood pressure (DBP) and pulse rate in a randomized, placebo-controlled, double-blind, crossover, dose-response study of 15 healthy subjects. Data are presented as mean ± SD. General linear model (GLM) with repeated measures was performed for comparison within and between treatment groups. Paired *t*-test was used for comparison of infusion period (at the beginning of LNMMA infusion period/at the end of LNMMA infusion period) vs baseline (prior to LNMMA infusion period) and LNMMA infusion period vs post infusion period (30 min after the end of LMMMA infusion period/60 min after the end of LNMMA infusion period). ^*^ *p* < 0.05. One-way ANOVA was performed to test differences between treatment groups. (PDF 356 kb)

## Abbreviations

AI: Augmentation index
bBP: brachial blood pressure
cBP: central blood pressure
C_H2O_: Free water clearance
EMA: European Medicines Agency.
FE_Na_: Fractional excretion of sodium
GFR (^51^Cr-EDTA-clearance): Glomerular filtration rate
L-NMMA: L-NG-monomethyl-arginine
NO: Nitric Oxide
p-Aldo: plasma aldosterone
p-AngII: plasma angiotensinII
p-AVP: plasma vasopressin
PRC: Plasma concentrations of rennin
PW: Pulse wave velocity
u-AQP2: urinary aquaporin-2 channels
u-ENaCγ: urinary epithelial sodium channels
UO: Urine output
