# Supplementary material for: Effect of tolvaptan on renal water and sodium excretion and blood pressure during nitric oxide inhibition: a dose-response study in healthy subjects
Source: BMC Nephrol. 2017 Mar 13;18:86. doi: 10.1186/s12882-017-0501-1 (PMC5347830; doi:10.1186/s12882-017-0501-1)
Supplement: Additional file 1: Table S1. — Urine output, free water clearance (CH2O), urinary AQP2 excretion per minute (u-AQP2), urinary sodium excretion (u-Na) and urinary potassium excretion (u-K) during 24-h urine collection in a randomised, placebo-controlled, double-blind, crossover, dose-response study of 15 healthy subjects. Values are means with ± SD. One-way ANOVA was used for comparison between groups. (PDF 9 kb) [file 12882_2017_501_MOESM1_ESM.pdf]

	Before each examination day				p (ANOVA)
	**Placebo**	**Tolvaptan 15 mg**	**Tolvaptan 30 mg**	**Tolvaptan 45 mg**	
**Urine Output (ml/24 h)**	2219 ± 750	2195 ± 485	2360 ± 684	2300 ± 543	0.88
**C_{H2O} (ml/min)**	-0.22± 0.58	-0.22± 0.51	-0.14± 0.50	-0.24± 0.53	0.99
**u-AQP2 (ng/min)**	0.93± 0.16	0.91± 0.12	0.96± 0.20	0.95± 0.18	0.66
**u-Na (mmol/24 h)**	102± 47	100± 36	101±35	103±36	0.90
**u-K (mmol/24h)**	52± 15	52± 12	46±13	53±17	0.74
