# Supplementary material for: Effect of tolvaptan on renal water and sodium excretion and blood pressure during nitric oxide inhibition: a dose-response study in healthy subjects
Source: BMC Nephrol. 2017 Mar 13;18:86. doi: 10.1186/s12882-017-0501-1 (PMC5347830; doi:10.1186/s12882-017-0501-1)
Supplement: Additional file 2: Table S2. — Effect of tolvaptan 15, 30 and 45 mg at baseline, during and after NO-inhibition on plasma concentration of sodium and plasma osmolality in a randomized, placebo-controlled, double-blind, crossover, dose-response study of 15 healthy subjects. Data are presented as mean ± SD. General linear model (GLM) with repeated measurements was performed for comparison within and between groups. One-way ANOVA was used to test differences between tolvaptan 15, 30 and 45 mg vs placebo. Paired t-test was performed for comparison of infusion period (90–150 min) vs baseline period (0–90 min), and post infusion period (150–210 min) vs baseline period. (PDF 84 kb) [file 12882_2017_501_MOESM2_ESM.pdf]

| Periods | Baseline | L-NMMA | | Post infusion | | p (GLM-within) |
|---|---|---|---|---|---|---|
| | 0-90 min | 90-120 min | 120-150 min | 150-180 min | 180-210 min | |
| **p-sodium (mmol/l)** | | | | | | |
| Placebo | 139± 1 | 139± 2 | 138± 2 | 138± 2 | 138± 2 | < 0.0001 |
| Tolvaptan 15 mg | 141± 1 | 141± 1 | 140 ± 1 | 141± 1 | 141± 2 | |
| Tolvaptan 30 mg | 141± 2 | 142± 2 | 142 ±2 | 142± 2 | 142± 2 | |
| Tolvaptan 45 mg | 141± 1 | 142± 2 | 141± 2 | 142± 2 | 142± 2 | |
| p (GLM between) | <0.0001 | | | | | |
| p (ANOVA) | 0.004 | < 0.0001 | < 0.0001 | < 0.0001 | < 0.0001 | |
| **p- osm (mosm/kg)** | | | | | | |
| Placebo | 285± 4 | 284± 4 | 283± 4 | 283± 5 | 281± 3 | < 0.0001 |
| Tolvaptan 15 mg | 288± 3 | 289± 3 | 288± 3 | 289± 3 | 288± 4 | |
| Tolvaptan 30 mg | 288± 3 | 290± 3 | 290± 3 | 290± 4 | 290± 3 | |
| Tolvaptan 45 mg | 288± 3 | 290± 3 | 289± 4 | 290± 4 | 290± 3 | |
| p (GLM between) | <0.0001 | | | | | |
| p (ANOVA) | 0.018 | <0.0001 | <0.0001 | <0.0001 | <0.0001 | |
