# Supplementary material for: Effect of tolvaptan on renal water and sodium excretion and blood pressure during nitric oxide inhibition: a dose-response study in healthy subjects
Source: BMC Nephrol. 2017 Mar 13;18:86. doi: 10.1186/s12882-017-0501-1 (PMC5347830; doi:10.1186/s12882-017-0501-1)
Supplement: Additional file 3: Table S3. — Effect of tolvaptan 15, 30 and 45 mg at baseline, during and after NO-inhibition on plasma concentrations of renin (PRC), angiotensin II (P-AngII) and aldosterone (P-aldo) in a randomized, placebo-controlled, double-blind, crossover, dose-response study of 15 healthy subjects. Data are presented as mean ± SD. General linear model (GLM) with repeated measures was performed for comparison within and between groups. Paired t-test was used for comparison between L-NMMA infusion period (at the end of L-NMMA infusion period) vs baseline (prior to L-NMMA infusion period) and at baseline vs post infusion period (1 h after L-NMMA infusion period) vs baseline period. One-way ANOVA was performed to test differences between treatment groups. (PDF 14 kb) [file 12882_2017_501_MOESM3_ESM.pdf]

Periods	Prior to L-NMMA infusion period	At the end of L-NMMA infusion period	1 hour after L-NMMA infusion period	P (GLM-within)
**PRC(pg/ml)**				
Placebo	5.8± 1.6	4.4± 1.5	4.1± 1.7	0.633
Tolvaptan 15 mg	6.1± 2.1	4.2± 2.1	4.7± 2.0	
Tolvaptan 30 mg	6.3± 1.8	4.4± 1.7	4.5± 1. 9	
Tolvaptan 45 mg	6.8± 1.8	4.9± 1.6	5.5± 1.5	
p (GLM between)		0.838		
**P-AngII (pg/ml)**				
Placebo	16.4± 2.3	13.5± 3.0	13.5± 2.9	0.864
Tolvaptan 15 mg	16.4± 2.6	12.2± 3.6	13.5± 3.3	
Tolvaptan 30 mg	13.5± 2.6	11.0± 1.8	11.0± 3.4	
Tolvaptan 45 mg	18.2± 2.2	14.9 ± 2.6	14.9± 2.7	
p (GLM between)		0.854		
**P- Aldo (pmol/L)**				
Placebo	69± 2	65 ± 2	59± 2	0.568
Tolvaptan 15 mg	67± 2	64± 2	67± 2	
Tolvaptan 30 mg	62± 2	63± 2	62± 2	
Tolvaptan 45 mg	69± 2	69± 2	69± 2	
p (GLM between)		0.999		
