# Supplementary material for: Effect of tolvaptan on renal water and sodium excretion and blood pressure during nitric oxide inhibition: a dose-response study in healthy subjects
Source: BMC Nephrol. 2017 Mar 13;18:86. doi: 10.1186/s12882-017-0501-1 (PMC5347830; doi:10.1186/s12882-017-0501-1)
Supplement: Additional file 4: Table S4. — Effect of tolvaptan 15, 30 and 45 mg at baseline, during and after NO-inhibition on brachial systolic blood pressure (SBP), diastolic blood pressure (DBP) and pulse rate in a randomized, placebo-controlled, double-blind, crossover, dose-response study of 15 healthy subjects. Data are presented as mean ± SD. General linear model (GLM) with repeated measures was performed for comparison within and between treatment groups. Paired t-test was used for comparison of infusion period (at the beginning of LNMMA infusion period/at the end of LNMMA infusion period) vs baseline (prior to LNMMA infusion period) and LNMMA infusion period vs post infusion period (30 min after the end of LMMMA infusion period/60 min after the end of LNMMA infusion period). * p < 0.05. One-way ANOVA was performed to test differences between treatment groups. (PDF 356 kb) [file 12882_2017_501_MOESM4_ESM.pdf]

| Periods | Baseline | L-NMMA | | Post infusion | | p (GLM-within) |
|---|---|---|---|---|---|---|
| | Prior to L-NMMA infusion | At the beginning of infusion | At the end of infusion | 30 min after the end of L-NMMA infusion | 60 min after the end of L-NMMA infusion | |
| **SBP (mmHG)** | | | | | | |
| Placebo | 113± 8 | 116± 8 | 116± 8 | 117± 9 | 118±10 | 0.179 |
| Tolvaptan 15 mg | 114± 8 | 118±8 | 117± 7 | 115± 6 | 117± 7 | |
| Tolvaptan 30 mg | 114± 9 | 116± 8 | 116± 8 | 117± 9 | 118± 9 | |
| Tolvaptan 45 mg | 114± 10 | 116± 7 | 117± 9 | 117± 8* | 119± 10 | |
| p (GLM between) | 0.993 | | | | | |
| **DBP (mmHg)** | | | | | | |
| Placebo | 62± 5 | 68± 7* | 68± 5 | 67± 6 | 66± 7 | 0.691 |
| Tolvaptan 15 mg | 62± 7 | 68± 7 | 67± 8 | 65± 6 | 66± 7 | |
| Tolvaptan 30 mg | 62± 6 | 68± 6 | 68± 7 | 67± 6 | 66± 6 | |
| Tolvaptan 45 mg | 61± 6 | 68± 6 | 68± 7 | 66± 8 | 67± 8 | |
| p (GLM between) | 0.997 | | | | | |
| **Pulse rate (BPM)** | | | | | | |
| Placebo | 57±9 | 52±10 | 53 ±8 | 56± 9 | 59 ±10 | 0.149 |
| Tolvaptan 15 mg | 58± 9 | 51±8 | 51±8 | 55±7 | 58±8 | |
| Tolvaptan 30 mg | 58±8 | 54±8 | 54±8 | 57±8 | 61±9 | |
| Tolvaptan 45 mg | 57± 8 | 53± 7 | 53 ± 8 | 55 ±8 | 59± 9 | |
| p (GLM between) | 0.906 | | | | | |
